# 

**Published:** 1861-08

**Authors:** 


### CHICAGO MEDICAL JOURNAL.

**Terms, $2.00 per Annum.**

### CONTENTS OF THE AUGUST NUMBER.

ORIGINAL COMMUNICATIONS.

PAGE.

Purulent Ophthalmia of Armies. By E. L. Holmes, M. D , of Chicago.... 443
Case of Amputation. By L. B. Brown, M. D., Iroquois, Ill.............. 448

SELECTED.

Directions to Army Surgeons on the Field of Battle. By G. J. Guthrie, Surgeon-General to the British Forces during the Crimean War... 450
Effects of Climate on Northern and Southern Constitutions.............. 455
Gunshot Wounds Produced during the Loading of Artillery.............. 457
On the Antagonistic Effects of Opium and Sulphate of Quinia. By Nelson Nivison, M. D., of Hector, Schuyler County, New York........... 458
Dr. David Skae's Definition of Insanity.................................. 467
Puerperal Insanity.............................................................. 468
Camp Dysentery. By C. D. Griswold, M. D., Cleveland, Ohio........... 471
Virchow's Pathological Views. By Dr. R. K. Browne, Professor of Physiology in the New York Medical College......................... 472

EDITORIAL.

Editorial and Miscellaneous.................................................. 492
Is Primary Chancre ever a Derivative of Secondary Syphilis by Inoculation........................................................................ 496

